# Vitamin D Status in Patients Operated for Primary Hyperparathyroidism: Comparison of Patients from Southern and Northern Europe

**DOI:** 10.1155/2013/164939

**Published:** 2013-08-05

**Authors:** Erik Nordenström, Antonio Sitges-Serra, Joan J. Sancho, Mark Thier, Martin Almquist

**Affiliations:** ^1^Department of Surgery, Lund University Hospital, 221 85 Lund, Sweden; ^2^Departement of Clinical Sciences, Lund University, Sweden; ^3^Department of Surgery, Skane University Hospital, Lund University, S-221 85 Lund, Sweden; ^4^Endocrine Surgery Unit, Department of Surgery, Barcelona, Spain

## Abstract

*Aim*. The interaction between vitamin D deficiency and primary hyperparathyroidism (PHPT) is not fully understood. The aim of this study was to investigate whether patients with PHPT from Spain and Sweden differed in vitamin D status and PHPT disease activity before and after surgery. *Methods*. We compared two cohorts of postmenopausal women from Spain (*n* = 126) and Sweden (*n* = 128) that had first-time surgery for sporadic, uniglandular PHPT. Biochemical variables reflecting bone metabolism and disease activity, including levels of 25-hydroxy vitamin D_3_ (25(OH)D) and bone mineral density, BMD, were measured pre- and one year postoperatively. *Results*. Median preoperative 25(OH)D levels were lower, and adenoma weight, PTH, and urinary calcium levels were higher in the Spanish cohort. The Spanish patients had higher preoperative levels of PTH (13.5 versus 11.0 pmol/L, *P* < 0.001), urinary calcium (7.3 versus 4.1 mmol/L, *P* < 0.001), and heavier adenomas (620 versus 500 g, *P* < 0.001). The mean increase in BMD was higher in patients from Spain and in patients with vitamin D deficiency one year after surgery. *Conclusion*. Postmenopasual women with PHPT from Spain had a more advanced disease and lower vitamin 25(OH)D levels. Improvement in bone density one year after surgery was higher in patients with preoperative vitamin D deficiency.

## 1. Introduction

Primary hyperparathyroidism (PHPT) is a common endocrine disorder affecting about 2-3% of all postmenopausal women [1] in Europe. PHPT and vitamin D deficiency coexist in elderly patients [2, 3]. The association between vitamin D and PHPT is complex and not fully understood [4–6]. It is not known if vitamin D deficiency could trigger PHPT or vitamin D deficiency is caused or worsened by PHPT. Patients with PHPT and low levels of 25-hydroxyvitamin D (25(OH)D) have a more severe bone disease [7–9]. Previous data has also suggested that suboptimal vitamin D nutrition is linked to parathyroid adenoma growth and that improved vitamin D nutrition might decrease PHPT disease activity [10]. 

Vitamin D status in general is influenced by sun exposure and nutrition, and data from previous studies have shown that vitamin D deficiency is common among elderly people both in northern and southern Europe [11, 12].

One study has actually shown that vitamin D deficiency is more common in southern than northern Europe indicating that not only the number of sun hours per year of a country is important in the context of vitamin D deficiency [13]. Comparisons between different cohorts of patients with PHPT from different part of Europe with assumed different sun exposure and/or different vitamin D nutrition are lacking. PHPT is associated with increased bone turnover and increased fracture risk [14, 15].

Thus, on average, BMD increases after successful surgery for pHPT, but not all patients experience improvement in BMD. One year after parathyroidectomy about half of the patients will have a significant remineralisation [16, 17]. Factors like the severity of primary hyperparathyroidism, age, and renal function has been found to influence bone recovery after surgery. 

The aim of this study was to investigate if two cohorts of postmenopausal women with primary hyperparathyroidism with assumed low sun exposure (Sweden) and high sun exposure (Spain) differed preoperatively in vitamin D status, disease activity, and outcome after surgery measured as change in BMD and biochemical variables one year after surgery.

## 2. Methods

Data collected prospectively in two cohorts of patients with primary hyperparathyroidism from Spain and Sweden, respectively, were compared preoperatively, and at one year follow-up. 

Informed consent for anonymous use of data was obtained from all patients.

All patients were postmenopausal and had successful first time surgery for sporadic PHPT. 

The diagnosis of PHPT was histologically proven in all patients. Part of the Swedish cohort has been described in a previous paper [16], and data from the Spanish cohort was recently published [17]. 

### 2.1. Clinical and Biochemical Variables

Demographic data and history of fractures up to 10 years before surgery were recorded. Serum levels of calcium, phosphate, alkaline phosphatase, creatinine, intact PTH, 25(OH)D and 1,25(OH)_2_D_3_, urinary calcium output over 24 h, and GFR were determined before and 1 year after parathyroidectomy. In the Swedish population high performance liquid chromatography, HPLC, was used for assessment of the concentration of serum 25(OH)D (reference range >75 nmol/L), and 1,25(OH)_2_D_3_ (reference range 10–60 ng/L) was measured with a radio-receptor assay (Incstar, Stillwater, MN, USA). Concentration of plasma parathyroid hormone (PTH) was analysed by an immunoradiometric assay for intact PTH (Hitachi Modular-E), (reference range 1.6–6.9 pmol/L). The analysis has a total CV of 5% at 7 pmol/L and 5% at 100 pmol/L. 

In the Spanish group intact PTH was analyzed by an immunoradiometric assay, (reference range 14–55 pg/mL), 25(OH)D with a chemiluminescence immunoassay, CLI, reference range 8–80 ng/mL and 1,25(OH)_2_D_3_ with a radioimmunoassay, (reference range 15–56 pg/mL). To convert from the conventional unit to the SI unit, we multiplied by the conversion factor 0.105 for PTH, 2.496 for 25(OH)D, and with 2.6 for 1,25(OH)_2_D_3_. In the Spanish cohort GFR was assessed according to the Modification of Diet in Renal Disease formula and in the Swedish cohort the iohexol clearance method was used [18]. Vitamin D deficiency was defined as 25(OH)D < 50 nmol/L [12, 18].

### 2.2. Bone Mineral Density

BMD was measured by dual-energy absorptiometry (DEXA) at three sites, the femoral neck, total hip, and lumbar spine. In the Spanish cohort the QDR 4500 SL (Hologic, Waltham, Massachusetts, USA) machine was used. This technique has an *in vivo* coefficient of variation, CV, of 1 per cent at the lumbar spine and 1.6 per cent at the femoral neck. In the Swedish cohort the Lunar Expert XL (Lunar Corp, Madison, Wis, USA) equipment was used, with an *in vivo* CV of 1 per cent. Results were expressed as grams per square centimetre and as age and gender specific standard deviations (*z* scores). BMD was measured preoperatively and one year after parathyroidectomy. A significant increase in BMD for an individual patient was calculated according to the formula 1.96 × *√*2 × CV. This formula generates the value that separates two independent samples with 95 per cent confidence. For an individual in the Spanish cohort, the BMD should increase by at least 3.7 per cent to be significant and for an individual in the Swedish cohort the corresponding figure was 2.8 per cent. Unfortunately  *t* score was not assessed in all the Swedish patients and therefore not shown. 

### 2.3. Surgery and Followup

Bilateral neck exploration or selective parathyroidectomy depending on preoperative localization studies was performed under general anaesthesia. All adenomas were confirmed by typical histological features. All patients were seen 2–6 weeks and 1 year after parathyroidectomy. At this time, BMD and biochemical variables were reassessed. In the Spanish cohort one gram of elemental calcium per day was prescribed during the follow-up period. In the Swedish cohort prescription of calcium and vitamin D was done on demand.

### 2.4. Statistics

Results are expressed as medians (interquartile range, IQR). Wilcoxon and Mann-Whitney *U* tests were used when comparing continuous variables between groups. For categorical data statistical significance was analysed by the chi squared test (*χ* ^2^). Statistical significance was set at *P* < 0.050. Data were analysed with STATA 11.0 (Stata corp., Texas, USA).

## 3. Results

### 3.1. Preoperative Data

254 patients with a median age of 65 (57–73) years were included in the study. The median serum calcium level was 2.74 (2.65–2.85) mmol/L, and the median serum intact PTH level was 12.0 (9.3–16.2) pmol/L. The median adenoma weight in all patients was 546 (300–1200) mg. 19 patients in the Spanish and 29 in the Swedish cohort had a history of fragility fracture before parathyroidectomy. 63 per cent of the Spanish and 39 per cent of the Swedish patients had biochemical vitamin D insufficiency preoperatively (*P* = 0.007). There was no difference in age at surgery or preoperative calcium levels, but median preoperative levels of PTH, phosphate, urinary calcium, ALP, GFR, creatinine, 1,25(OH)_2_D_3_, and adenoma weight were all higher, and median preoperative 25(OH)D lower, in the Spanish cohort (see Table 1).

### 3.2. Followup One Year after Surgery

At one year after surgery, all patients in both cohorts remained normocalcemic. However, median levels of 1,25(OH)_2_D_3_ were higher, and median levels of calcium and 25(OH)D were lower, in the Spanish cohort as compared to the Swedish (see Table 2).

BMD increased one year after surgery as compared to preoperatively at all sites in both cohorts, both in absolute terms (g/cm^2^) and as *z*-scores (see Table 3). Preoperatively, BMD was higher in the Swedish cohort for lumbar spine and femoral neck in absolute terms, but not as *z*-scores. 

When the two cohorts were compared regarding change in BMD, the Spanish cohort improved more at the lumbar spine, in absolute terms, and at the femoral neck, as *z*-score. For the other measurements, there was no difference between the two cohorts (see Table 4).

### 3.3. Vitamin D Status

Bone remineralisation after operation in patients with or without vitamin D deficiency (25(OH)D < 50 nmol/L) are shown in Table 5. Patients with vitamin D deficiency had higher remineralization one year after surgery at total hip compared to patients with sufficient vitamin D levels, but not at the lumbar spine and/or femoral neck.

## 4. Discussion

In this study comparing postmenopausal Swedish and Spanish patients operated on for PHPT, the Spanish cohort had lower median levels of 25(OH)D whereas median PTH and urinary calcium levels were higher before surgery, compared to the Swedish group of patients. Median adenoma weight was also higher in the Spanish cohort, but preoperative calcium levels and age were similar in the two cohorts.

One year after surgery, we found no difference in median PTH levels between the two cohorts, but median levels of 25(OH)D were still lower in the Spanish than in the Swedish cohort, even though median levels of 25(OH)D were higher after surgery as compared to preoperatively in both cohorts. 

A potential explanation for these findings could be a selection bias, that is, that patients in Spain are referred and accepted for surgery in a more advanced stage of the disease. If this is the case, the lower levels of 25(OH)D in the Spanish cohort preoperatively might be due to increased conversion of 25(OH)D into 1,25(OH_2_)D_3_ by PTH-mediated renal 1*α*-hydroxylation [19]. This phenomenon is supported by the fact that PTH and 25(OH)D are inversely correlated; thus, in a more severe PHPT disease higher PTH and lower 25(OH)D are expected [17]. Patients with PHPT might also have low levels of 25(OH)D because of poor nutrition and/or less sun exposure [20]. Whether the high serum calcium and PTH levels may interfere with the conversion of 7-dehydrocholesterol in the skin by UV-B to previtamin D, or with the hydroxylation of vitamin D in the liver to 25(OH)D remains to be determined. 

We assumed that patients from Spain with PHPT had higher sun exposure than their Swedish counterparts [8]. However, sun exposure is not the only determinant of 25(OH)D levels. Hence, previous studies have shown a north-south gradient regarding serum 25(OH)D, with higher levels of 25(OH)D in Scandinavia and lower levels in Italy and Spain countries. This points to other determinants than sunshine, for example, nutrition, food fortification, and supplement use [13]. Unfortunately, information about nutrition was lacking in the present study. Thus, we cannot rule out that the Swedish patients were more inclined to take vitamin D supplementation and had a higher nutritional intake of vitamin D.

On the other hand the HELENA study has demonstrated that the winter half-year adolescents in Southern Europe have higher serum 25OHD levels compared to adolescents in Central Europe [21], so data about vitamin D status in different populations even without PHPT is conflicting.

Few studies have compared differences in preoperative status and outcome after PHPT surgery in two cohorts living in two countries that differ in distance from the equator, that is, different sun exposure and difference in preoperative D vitamin status [22, 23].

Silverberg compared two cohorts of patients with PHPT from New York City and Beijing, China. Patients from China had a more advanced PHPT disease and a more profound Vitamin D deficiency [23]. This could reflect the same findings we found between Swedish and Spanish patients, that is, patients from Spain and China come to surgery in a more advanced state of the PHPT disease.

However, in our study these mechanisms cannot account for the observed difference in 25(OH)D levels between the two cohorts after surgery, since at this point, both cohorts had similar levels of PTH and were normocalcemic, that is, cured of PHPT. Another explanation might instead be that low levels of 25(OH)D causes or at least worsens PHPT. One study suggests that low levels of 25(OH)D stimulate parathyroid adenoma growth [10]. 

It seems that it is very hard to define the mechanisms of interaction between vitamin D and PHPT exactly. We believe that the PHPT disease itself could worsen vitamin D deficiency but also the reverse situation, that is, vitamin D deficiency could worsen the PHPT disease.

Patients from Spain had preoperatively lower bone mineral density in lumbar spine and femoral neck probably because Spanish patients had a more advanced PHPT disease. The Spanish patients also gained more from the operation in terms of bone density suggesting that the remineralisation response is more pronounced in patients with lower BMD before parathyroidectomy [18]. 

Patients with vitamin D deficiency had higher increase in bone density in total hip. Previous studies have shown that patients with PHPT and vitamin D deficiency gain more in bone density after PHPT surgery [23]. This highlights the possible benefit of vitamin D supplementation in PHPT disease regarding gain in bone density. So far no convincing study has shown if postoperative vitamin D supplementation enhances increase in bone density after parathyroidectomy [9], and further studies are warranted.

The cohorts in the present study were homogeneous, and data was recorded in a prospective and consecutive manner. A limitation is that 25(OH)D was measured with different methods in Sweden and Spain, respectively. Previous results suggest that 25(OH)D levels measured with CLI, that is, the Spanish method in the present study yields lower levels than HPLC, that is, the Swedish method [24] Thus, the difference in 25(OH)D between the cohorts might be spurious and caused by the use of different methods. However, the other biochemical findings and bone density measurements support the conclusion that the Spanish patients suffered from a more severe PHPT disease. Another potential limitation is that we did not record season of blood sampling before PHPT surgery. However, there was no reason to suspect that distribution of season of operation differed between cohorts. Unfortunately, we did not have information about nutrition in the two cohorts. 

In conclusion we found that postmenopausal women with PHPT from Spain had lower preoperative levels of 25(OH)D and more severe PHPT compared to Swedish patients, and patients with PHPT and vitamin D deficiency gained more in bone density at some sites one year after parathyroidectomy.

## Figures and Tables

**Table 1:** Pre- and perioperative biochemical and clinical variables in patients operated on for primary hyperparathyroidism. Median, (IQR). Groups compared with Kruskal-wallis and chi square where appropriate.

Variable	All	Spanish cohort *n* = 126	Swedish cohort *n* = 128	*P *
Age (years)	65 (57–73)	64 (57–72)	66 (58–74)	*0.33 *
p-Ca (mmol/L)	2.74 (2.65–2.85)	2.74 (2.65–2.85)	2.74 (2.66–2.84)	*0.73 *
PTH (pmol/L)	12 (9.3–16.2)	13.5 (10.5–20.4)	11.0 (8.4–14.0)	*<0.001 *
25-OH-D (nmol/L)	44 (26–64)	40 (25–58)	51 (28–66)	*0.01 *
1,25-OH-D (pmol/L)	55 (98–162)	142 (99–198)	56 (28–66)	*<0.001 *
Phosphate (mmol/L)	0.85 (0.77–0.96)	0.90 (0.80–1.0)	0.82 (0.72–0.90)	*<0.001 *
ALP (unit)	2.6 (1.6–3.8)	3.0 (1.7–4.3)	2.3 (1.5–3.4)	*0.002 *
GFR (mL/min)	75 (63–89)	70 (58–85)	76 (65–93)	*0.01 *
Creatinine (umol/L)	71 (62–81)	80 (71–88)	65 (56–73)	*<0.001 *
Urinary calcium (unit)	5.9 (3.6–9)	7.3 (3.9–10.0)	4.1 (2.8–5.9)	*<0.001 *
Fracture (yes/no)	48/195	19/107	29/99	*0.002 *
Vitamin D deficiency (yes/no)	130/96	80/42	50/54	*0.007 *
Adenoma weight (mg)	546 (300–1200)	620 (290–1400)	500 (320–1030)	*<0.001 *

**Table 2:** Biochemical variables one year after surgery for primary hyperparathyroidism.

Variable	Spanish cohort *n* = 126	Swedish cohort *n* = 128	*P *
p-Ca (mmol/L)	2.32 (2.24–2.42)	2.37 (2.30–2.48)	*0.002 *
PTH (pmol/L)	4.7 (3.8–6.9)	4.7 (3.7–6)	*0.51 *
25-OH-D (nmol/L)	45 (24–65)	62 (49–84)	*<0.001 *
1,25-OH-D (pmol/L)	84 (0–120)	31 (38–50)	*<0.001 *
ALP	1.2 (0.9–2.5)	1.3 (1.0–2.1)	*0.35 *

**Table 3:** Bone mineral density before and one year after surgery for primary hyperparathyroidism. Medians (IQR) Wilcoxon's signed rank test.

BMD site	Spanish cohort Before surgery	Spanish cohort One year postop	*P *	Swedish cohort Before surgery	Swedish cohort One year postop	*P *	*P* diff Spanish-swedish preop
Lumbar spine, absolute	0.79 (0.70–0.88)	0.83 (0.74–0.93)	*<0.001 *	1.00 (0.83–1.11)	1.03 (0.88–1.14)	*<0.001 *	*<0.001 *
Lumbar spine, *z*-score	−0.7 (−1.6–0.3)	0.7 (0.4–1.3)	*<0.001 *	−0.4 (−1.6–0.6)	0.7 (0.4–1.38)	*<0.001 *	*0.75 *
Femoral neck, absolute	0.62 (0.56–0.69)	0.64 (0.57–0.70)	*<0.001 *	0.76 (0.69–0.85)	0.79 (0.70–0.88)	*<0.001 *	*<0.001 *
Femoral neck, *z*-score	−0.7 (−1.3 to −0.1)	−0.4 (−1.1–0.2)	*<0.001 *	−0.5 (−1.1–0.1)	−0.5 (−0.9–0.2)	*<0.001 *	*0.11 *
Total hip, absolute	0.76 (0.68–0.85)	0.76 (0.70–0.87)	*<0.001 *	0.77 (0.56–0.88)	0.84 (0.75–0.95)	*<0.001 *	*0.40 *
Total hip, *z*-score	−0.5 (−0.9–0.3)	−0.2 (−0.8–0.5)	*<0.001 *	−0.4 (1.0–0.4)	0 (−0.7–0.7)	*<0.001 *	*0.91 *

**Table 4:** Change in bone mineral density (Δ) after surgery for primary hyperparathyroidism. Change in absolute values in percent. Change in *z*-score in arithmetic difference. Medians (IQR). Kruskal-Wallis test.

BMD site	Spanish cohort	Swedish cohort	*P *
ΔLumbar spine, absolute	4.2 (1.0–6.9)	1.3 (−0.9–6.8)	*0.009 *
ΔLumbar spine, *z*-score	0.4 (0.2–0.6)	0.2 (0.0–0.6)	*0.18 *
ΔFemoral neck, absolute	2.8 (−0.9–7.3)	2.1 (−1.1–5.8)	*0.46 *
ΔFemoral neck, *z*-score	0.3 (0.0–0.5)	0.1 (−0.1–0.4)	*0.004 *
ΔTotal hip, absolute	2.8 (−0.3–5.7)	1.9 (0.6–4.2)	*0.09 *
ΔTotal hip *z*-score	0.3 (0.1–0.5)	0.2 (0.1–0.4)	*0.05 *

**Table 5:** Bone remineralisation after operation for primary hyperparathyroidism in patients with or without vitamin D deficiency (25-OH-D < 50 nmol/L). Change in absolute values in percent. Change in *z*-score in arithmetic difference. Medians (IQR). Kruskal-Wallis test.

BMD site	Vitamin D-deficiency	Vitamin D-sufficiency	*P *
	(25-OH-D < 50 nmol/L)	(25-OH-D ≥ 50 nmol/L)	
ΔLumbar spine, absolute	3.5 (−0.5–7.0)	2.2 (−2.0–6.6)	*0.12 *
ΔLumbar spine, *z*-score	0.4 (0.1–0.6)	0.3 (0.0–0.6)	*0.40 *
ΔFemoral neck, absolute	2.8 (−0.8–7.3)	2.5 (−1.9–6.0)	*0.47 *
ΔFemoral neck, *z*-score	0.3 (0–0.5)	0.2 (−0.1–0.3)	*0.05 *
ΔTotal hip, absolute	2.7 (0–5.7)	1.00 (−1.4–4.8)	*0.03 *
ΔTotal hip, *z*-score	0.3 (0.1–0.5)	0.2 (0.0–0.4)	*0.01 *
